# Rare Case of an Intramuscular Hemangioma of the Foot: A Case Report With a Review of the Literature

**DOI:** 10.7759/cureus.68711

**Published:** 2024-09-05

**Authors:** Lyubomir Gaydarski, Kristina Petrova, Boycho Landzhov, Georgi P Georgiev

**Affiliations:** 1 Department of Anatomy, Histology and Embryology, Medical University of Sofia, Sofia, BGR; 2 Clinical Laboratory, Medical University of Sofia, Sofia, BGR; 3 Orthopaedics and Traumatology, University Hospital "Queen Giovanna-ISUL", Sofia, BGR

**Keywords:** diagnostics, foot, histopathology (hp), intramuscular hemangioma, treatment

## Abstract

Hemangiomas are benign tumors characterized by an abnormal proliferation of blood vessels, which can be particularly challenging to diagnose and manage when located in unusual sites such as the foot. Herein, we report a case of a 36-year-old woman with a plantar hemangioma on the right foot, characterized by a long-standing, periodically changing subcutaneous lump. Clinical examination and magnetic resonance imaging revealed a hyperintense mass involving the musculus flexor digitorum brevis. The patient underwent surgical excision, which was complicated by intraoperative rupture of the mass but ultimately resulted in complete removal. Histopathological analysis confirmed the diagnosis of an intramuscular hemangioma. Postoperative recovery was uneventful, and follow-up showed no recurrence after six months. This case highlights the critical role of accurate diagnosis through physical examination and imaging, particularly magnetic resonance imaging, to differentiate benign hemangiomas from malignant tumors and guide treatment. While surgical excision is the primary treatment for symptomatic or cosmetically concerning hemangiomas, less invasive alternatives like sclerotherapy may be appropriate for superficial lesions. Effective management requires precise diagnostic imaging and a tailored therapeutic approach.

## Introduction

Hemangiomas are benign tumors composed of an abnormal proliferation of blood vessels. They exhibit a slow growth pattern, although they may become more prominent due to trauma or growth acceleration, particularly in children [1]. While malignant transformation is rare [1], hemangiomas can present significant clinical challenges depending on their location and size. Hemangiomas can be categorized into intramuscular hemangiomas (IMHs) and cutaneous hemangiomas. IMHs typically appear in adults, often before age 30, whereas cutaneous hemangiomas are more common in the pediatric population [2]. There is some debate about gender predisposition, with some studies indicating a female predominance, while others find no significant gender differences [3]. IMHs are relatively rare, with a prevalence of less than 1% [4]. When they occur, they are most frequently found in the legs, particularly in the thigh muscles, with foot localization notably rare [3]. IMHs often present as a palpable mass, which can cause local swelling and pain or remain asymptomatic.

Diagnostic approaches typically involve a thorough physical examination, magnetic resonance imaging (MRI), and histological examination following surgical excision [5]. MRI is particularly useful in delineating the extent of the tumor and its relation to surrounding structures. The primary treatment for IMHs is surgical excision. However, conservative methods such as sclerotherapy and steroid injections may also be considered depending on the hemangioma's size, location, and symptoms [6]. The current report presents the case of a 36-year-old female with plantar IMH on the right foot. In addition, we do a brief literature review and present the diagnosis, imaging, pathological findings, and surgical treatment of IMH of the foot.

## Case presentation

**Figure 1:**
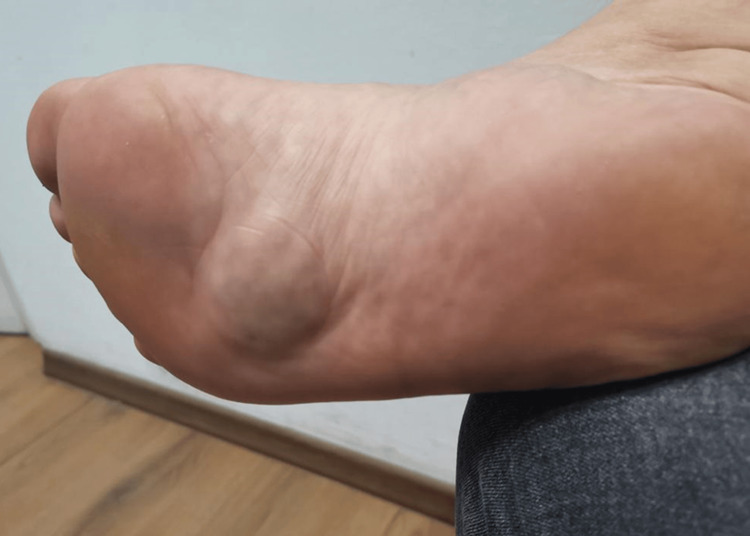
Preoperative photograph illustrating the tumorous mass on the plantar surface of the patient's right foot.

A 36-year-old female presented with a history of a subcutaneous lump on her right foot, which she reported had been present for 20 years. The mass exhibited periodic changes in size, increasing and decreasing intermittently. The patient's medical history was unremarkable, with no known comorbidities or allergies. Upon physical examination, a distinct tumor-like formation was identified: A soft, elastic mass approximately the size of a walnut (2 x 2 cm) was located on the plantar surface near the metatarsophalangeal joints of the right foot (Figure 1).

**Figure 2:**
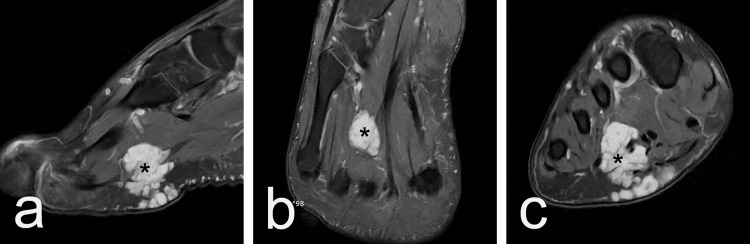
MRI illustrating a hyperintense mass in front of the bodies of the third and fourth metatarsal bones, involving the musculus flexor digitorum brevis. (a) View in the sagittal plane; (b) view in the transverse plane; (c) view in the frontal plane.

MRI revealed a hyperintense mass located anterior to the bodies of the third and fourth metatarsal bones. The mass measured 202 × 212 mm and appeared to involve the musculus flexor digitorum brevis (Figure 2). 

**Figure 3:**
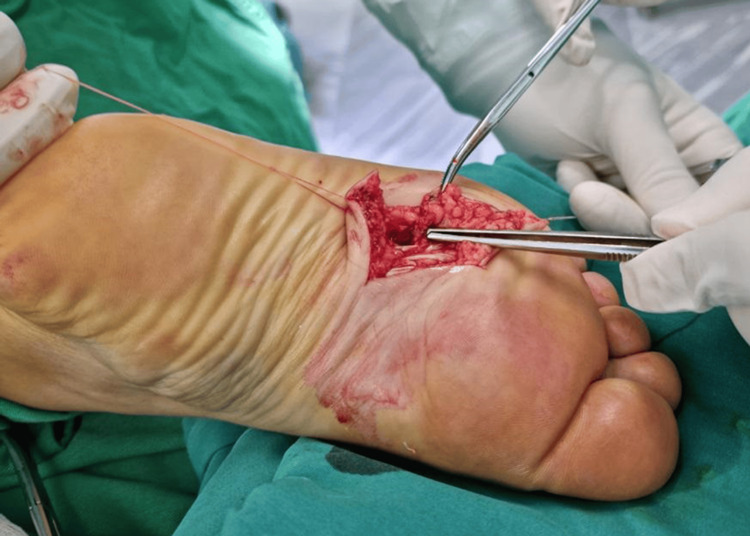
Intraoperative photograph depicting the extirpation of the tumor. The tip of the tweezers indicates the location where the tumor mass ruptured, resulting in the drainage of dark blood.

Based on clinical and imaging findings, a diagnosis of benign neoplasms in front of the metatarsal bones of the right foot was established. The patient subsequently underwent surgical excision of these masses. A Z-shaped skin incision was made, and the mass was meticulously separated from the surrounding tissue. However, the mass ruptured and drained, complicating the complete removal of the tumor. Nevertheless, the surgery was successfully completed, and the tumor was entirely extirpated from the plantar region of the right foot (Figure 3). 

**Figure 4:**
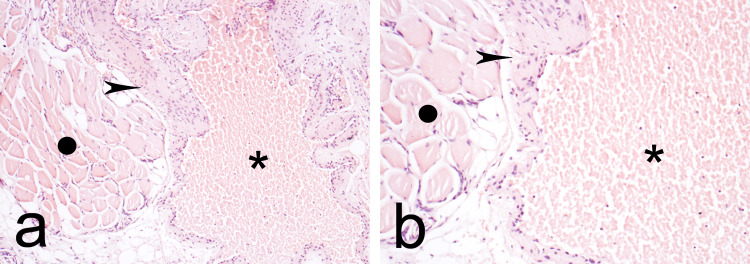
Histopathological photograph showcasing the typical features of a cavernous intramuscular hemangioma at (a) magnification ×10 and (b) magnification ×40. The black circle indicates muscle tissue; the arrowhead points to a fibrous septum outlined with endothelium; and the asterisks (*) indicate the cavernous space filled with blood.

The histopathological assessment confirmed the diagnosis of IMH by revealing typical histological features consistent with the cavernous subtype of an IMH. Such features include cavernous-like vessels, fibrous septs outlined with epithelioid endothelial cells, muscle cells, and adipocytes (Figure 4). 

**Figure 5:**
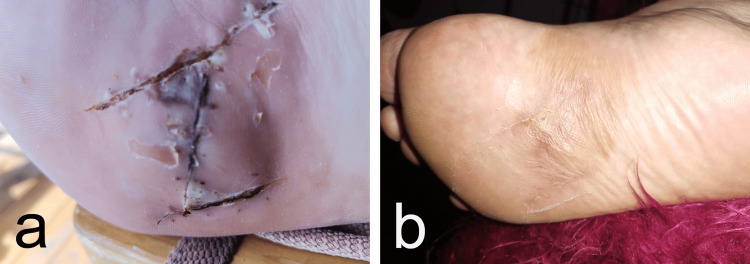
Postoperative photographs taken 14 days after surgery (a) and at the six-month follow-up (b).

The postoperative course was uneventful, and the patient was discharged in a stable and improved condition. Follow-up evaluations conducted at 14 days (Figure 5a) and six months postoperatively showed no evidence of recurrence (Figure 5b). The patient reported no clinical complaints, indicating a satisfactory outcome. 

## Discussion

Herein, we present a complex case of IMH of the foot with thoroughly presented diagnostic and treatment. Skeletal muscle hemangiomas are uncommon but crucial to consider when diagnosing unexplained soft tissue masses or pain. Early comprehensive research by Allen and Enziger explored these tumors in detail, analyzing 89 cases to describe their clinical manifestations and histological characteristics [7]. Their work laid the groundwork for understanding these benign vascular tumors, which can be challenging to differentiate from malignant conditions based solely on clinical presentation [7].

The evaluation of IMHs has revealed significant clinical challenges and diagnostic complexities, with various studies contributing to a deeper understanding of these lesions. Cohen et al. emphasized the frequent presentation of these tumors as soft tissue masses, often necessitating extensive diagnostic workups to distinguish them from malignancies [8]. Yuh et al. highlighted the critical role of MRI, particularly the observation of hyperintense lesions on T2-weighted images, in differentiating benign IMHs from other soft tissue pathologies [9]. Jenner et al. further contributed by examining MRI characteristics in 16 cases of skeletal muscle hemangiomas, offering comprehensive insights into their imaging features [10].

Our study provides additional insights into the radiological appearance of IMH. The clinical management of IMHs has been enriched by detailed case reports, such as that by Nack and Gustafson, who presented an in-depth clinical presentation and review of the available treatment options at that time [11]. Wild et al. documented additional cases, which helped to clarify the clinical and imaging profiles of IMHs [12]. Moreover, Lamm et al. described a 15-year-old male with a long-standing painful lump on his foot, diagnosed via MRI as a cavernous hemangioma, who opted for conservative management with insoles, despite the recommendation for surgery [13]. Similarly, Wisniewski et al. reported a case of a 16-year-old dancer initially misdiagnosed with plantar fasciitis, later identified through MRI to have an IMH in the flexor digitorum brevis muscle; sclerotherapy was chosen over surgery, yet the patient continued to experience moderate pain [14]. Mitsionis et al. discussed a case of a 12-year-old boy with a palpable foot mass that was successfully excised, allowing for a smooth recovery [4].

The possible treatment approaches are further illustrated by Uslu et al., who reported on two cases: a seven-year-old girl with an IMH that was surgically removed with no recurrence and a nine-year-old boy who required sclerotherapy and subsequent additional treatment for symptom resolution [5]. Furthermore, Lahrach et al. reported a case involving a 24-year-old man with a long history of forefoot pain and a firm mass, which was surgically excised with a full recovery [6]. In addition, Boedijono et al. described an 18-year-old woman with a recurrent IMH, successfully treated with wide-margin surgical excision [3]. Lastly, Lee et al. reported on a 25-year-old woman with chronic foot pain, where an MRI identified an IMH within the flexor digitorum brevis muscle, and surgical intervention led to significant pain relief with no recurrence [15]. Collectively, these studies underscore the importance of MRI in diagnosing IMHs and demonstrate the varied yet successful treatment strategies, ranging from conservative management to surgical excision, which can lead to positive outcomes. Our study shares several similarities with the discussed research, yet it further enriches the existing literature on diagnosing and treating IMH. The discussed cases are summarized in Table 1.

**Table 1:** Summary of cases of plantar intramuscular hemangiomas.

	Age	Sex	Diagnostic method	Location	Measurements	Symptoms	Treatment
Boedijono DR [3]	18	Female	MRI	Flexor digitorum brevis and abductor hallucis muscles of the left foot	N/A	Pain and swelling	Surgery
Mitsionis GI et al. [4]	12	Male	MRI	Flexor digitorum brevis muscle of the right foot	2.4 x 1.3 x 1.2 cm	Pain and swelling	Surgery
Uslu M et al. [5]	7	Female	MRI	Flexor digitorum brevis muscle of the right foot	4 x 3 x 5 cm	Pain and swelling	Surgery
Uslu M et al. [5]	9	Male	MRI, Doppler Ultrasound, Phlebography	Plantar hemagioma of the left foot	N/A	Pain and swelling	Sclerotherapy
Lahrach K et al. [6]	24	Male	X-ray, MRI	First intermetatarsal space of the left foot	30 × 22 mm	Pain	Surgery
Lamm BM, Pontious J [13]	15	Male	MRI	Plantar hemagioma of the left foot	5.5 × 3.0 × 2.3 cm	Pain	Conservative
Wisniewski SJ et al. [14]	16	Female	MRI, Angiography	Flexor digitorum brevis muscle of the left foot	2 x 2.3 x 4 cm	Pain and swelling	Sclerotherapy
Lee HS et al. [15]	25	Female	X-ray, MRI	Flexor digitorum brevis and plantar fascia of the right foot	5.6 × 2.8 × 2.5 cm	Pain and swelling	Surgery

Differentiating hemangiomas from other soft tissue masses is vital for appropriate management, as emphasized by Damron et al. [16]. Their comprehensive review of various soft-tissue abnormalities underscores the critical role of accurate diagnosis in determining effective treatment strategies. Suh et al. [17] further advanced diagnostic criteria by detailing MRI findings in a cohort of 23 patients with soft tissue tumors, refining the criteria for identifying hemangiomas. Treatment approaches for hemangiomas have evolved significantly. Tang et al. [18] discussed the role of surgical intervention, particularly for lesions that cause considerable symptoms or aesthetic concerns. Their study highlights that while surgical excision often results in favorable outcomes, the potential for recurrence remains a notable risk. Sclerotherapy has emerged as an essential alternative, especially for superficial or cosmetically problematic hemangiomas. Winter et al. [19] provided insights into this method, which involves the injection of sclerosing agents to destroy the vascular components of the tumor, presenting a less invasive option than surgery. The utility of MRI extends beyond diagnosis to include therapeutic monitoring. Hayashi et al. explored the application of MR-guided sclerotherapy, a technique that leverages MRI to enhance treatment efficacy and minimize tissue damage. Monitoring changes in signal intensity during the procedure ensures more precise and effective management of hemangiomas [20].

## Conclusions

This study and the associated literature review emphasize the diagnostic and therapeutic challenges of IMHs. The presented case of a 36-year-old woman with plantar IMH underscores the importance of accurate diagnosis through physical examination and MRI, which are crucial for differentiating these benign tumors from malignancies. MRI has proven essential in identifying the characteristic features of IMHs and guiding treatment. While surgical excision remains a primary method for managing symptomatic or aesthetically problematic lesions, sclerotherapy offers a less invasive alternative, especially for superficial hemangiomas. Advances in MRI-guided sclerotherapy further enhance treatment precision. Overall, effective management of IMHs relies on accurate diagnosis and a tailored approach to treatment, with ongoing research needed to refine these strategies and improve patient outcomes.
